# Proceedings of the First Curing Coma Campaign NIH Symposium: Challenging the Future of Research for Coma and Disorders of Consciousness

**DOI:** 10.1007/s12028-021-01260-x

**Published:** 2021-07-08

**Authors:** Jan Claassen, Yama Akbari, Sheila Alexander, Mary Kay Bader, Kathleen Bell, Thomas P. Bleck, Melanie Boly, Jeremy Brown, Sherry H.-Y. Chou, Michael N. Diringer, Brian L. Edlow, Brandon Foreman, Joseph T. Giacino, Olivia Gosseries, Theresa Green, David M. Greer, Daniel F. Hanley, Jed A. Hartings, Raimund Helbok, J. Claude Hemphill, H. E. Hinson, Karen Hirsch, Theresa Human, Michael L. James, Nerissa Ko, Daniel Kondziella, Sarah Livesay, Lori K. Madden, Shraddha Mainali, Stephan A. Mayer, Victoria McCredie, Molly M. McNett, Geert Meyfroidt, Martin M. Monti, Susanne Muehlschlegel, Santosh Murthy, Paul Nyquist, DaiWai M. Olson, J. Javier Provencio, Eric Rosenthal, Gisele Sampaio Silva, Simone Sarasso, Nicholas D. Schiff, Tarek Sharshar, Lori Shutter, Robert D. Stevens, Paul Vespa, Walter Videtta, Amy Wagner, Wendy Ziai, John Whyte, Elizabeth Zink, Jose I. Suarez, the Curing Coma Campaign

**Affiliations:** 1 Department of Neurology, Columbia University and New York-Presbyterian Hospital, 177 Fort Washington Avenue, MHB 8 Center, Room 300, New York City, NY 10032 USA; 2 Departments of Neurology, Neurological Surgery, and Anatomy & Neurobiology and Beckman Laser Institute and Medical Clinic, University of California, Irvine, Irvine, CA USA; 3 Acute and Tertiary Care, School of Nursing and Critical Care Medicine, School of Medicine, University of Pittsburgh, Pittsburgh, PA USA; 4 Mission Hospital, Mission Viejo, CA USA; 5 Department of Physical Medicine and Rehabilitation, University of Texas Southwestern Medical Center, Dallas, TX USA; 6 Davee Department of Neurology, Feinberg School of Medicine, Northwestern University, Chicago, IL USA; 7 Department of Neurology, School of Medicine and Public Health, University of Wisconsin–Madison, Madison, WI USA; 8 Office of Emergency Care Research, Division of Clinical Research, National Institute of Neurological Disorders and Stroke, Bethesda, MD USA; 9 Departments of Critical Care Medicine, Neurology, and Neurosurgery, School of Medicine, University of Pittsburgh, Pittsburgh, PA USA; 10 Department of Neurology, Washington University in St. Louis, St. Louis, MO USA; 11 Department of Neurology, Center for Neurotechnology and Neurorecovery, Massachusetts General Hospital and Harvard Medical School, Harvard University, Boston, MA USA; 12 Departments of Neurology and Rehabilitation Medicine, College of Medicine, University of Cincinnati, Cincinnati, OH USA; 13 Department of Physical Medicine and Rehabilitation, Harvard Medical School, Harvard University, Boston, MA USA; 14 GIGA Consciousness After Coma Science Group, University of Liege, Liege, Belgium; 15 School of Nursing, Queensland University of Technology, Kelvin Grove, QLD Australia; 16 Department of Neurology, School of Medicine, Boston University, Boston, MA USA; 17 Division of Brain Injury Outcomes, Department of Neurology, School of Medicine, Johns Hopkins University, Baltimore, MD USA; 18 Department of Neurosurgery, College of Medicine, University of Cincinnati, Cincinnati, OH USA; 19 Neurocritical Care Unit, Department of Neurology, Medical University of Innsbruck, Innsbruck, Austria; 20 Department of Neurology, Weill Institute for Neurosciences, School of Medicine, University of California, San Francisco, San Francisco, CA USA; 21 Department of Neurology, School of Medicine, Oregon Health & Science University, Portland, OR USA; 22 Department of Neurology, Stanford University, Palo Alto, CA USA; 23 Department of Pharmacy, Barnes Jewish Hospital, St. Louis, MO USA; 24 Departments of Anesthesiology and Neurology, Duke University, Durham, NC USA; 25 Department of Clinical Medicine, University of Copenhagen, Copenhagen, Denmark; 26 Department of Neurology, Rigshospitalet, Copenhagen University Hospital, Copenhagen, Denmark; 27 College of Nursing, Rush University, Chicago, IL USA; 28 Center for Nursing Science, University of California, Davis, Sacramento, CA USA; 29 Department of Neurology, The Ohio State University, Columbus, OH USA; 30 Department of Neurology, New York Medical College, Valhalla, NY USA; 31 Interdepartmental Division of Critical Care, Department of Respirology, University of Toronto, Toronto, ON Canada; 32 College of Nursing, The Ohio State University, Columbus, OH USA; 33 Department of Intensive Care Medicine, University Hospitals Leuven and University of Leuven, Leuven, Belgium; 34 Departments of Neurosurgery and Psychology, Brain Injury Research Center, University of California, Los Angeles, Los Angeles, CA USA; 35 Departments of Neurology, Anesthesiology/Critical Care, and Surgery, Medical School, University of Massachusetts, Worcester, MA USA; 36 Department of Neurology, Weill Cornell Medical College, New York City, NY USA; 37 Division of Neurosciences Critical Care, Departments of Anesthesiology and Critical Care Medicine, Neurology, and Neurosurgery, School of Medicine, Johns Hopkins University, Baltimore, MD USA; 38 Departments of Neurology and Neurosurgery, University of Texas Southwestern Medical Center, Dallas, TX USA; 39 Departments of Neurology and Neuroscience, School of Medicine, University of Virginia, Charlottesville, VA USA; 40 Department of Neurology, Harvard Medical School, Harvard University, Boston, MA USA; 41 Department of Neurology, Albert Einstein Israelite Hospital and Universidade Federal de São Paulo, São Paulo, Brazil; 42 Department of Biomedical and Clinical Sciences “L. Sacco”, Università degli Studi di Milano, Milan, Italy; 43 Department of Neurology and Brain Mind Research Institute, Weill Cornell Medicine, Cornell University, New York City, NY USA; 44 Department of Intensive Care, Paris Descartes University, Paris, France; 45 Departments of Neurosurgery and Neurology, University of California, Los Angeles, Los Angeles, CA USA; 46 National Hospital Alejandro Posadas, Buenos Aires, Argentina; 47 Department of Physical Medicine and Rehabilitation, School of Medicine, University of Pittsburgh, Pittsburgh, PA USA; 48 Moss Rehabilitation Research Institute, Elkins Park, PA USA; 49 Division of Neurosciences Critical Care, Department of Neurology, School of Medicine, Johns Hopkins University, Baltimore, MD USA

**Keywords:** Coma, Consciousness, Electrophysiology, Magnetic resonance imaging, Biomarkers

## Abstract

Coma and disorders of consciousness (DoC) are highly prevalent and constitute a burden for patients, families, and society worldwide. As part of the Curing Coma Campaign, the Neurocritical Care Society partnered with the National Institutes of Health to organize a symposium bringing together experts from all over the world to develop research targets for DoC. The conference was structured along six domains: (1) defining endotype/phenotypes, (2) biomarkers, (3) proof-of-concept clinical trials, (4) neuroprognostication, (5) long-term recovery, and (6) large datasets. This proceedings paper presents actionable research targets based on the presentations and discussions that occurred at the conference. We summarize the background, main research gaps, overall goals, the panel discussion of the approach, limitations and challenges, and deliverables that were identified.

**Supplementary Information:**

The online version contains supplementary material available at 10.1007/s12028-021-01260-x.

## Introduction

Coma and disorders of consciousness (DoC) result from a wide range of etiologies and are prevalent worldwide. In 2019, the Neurocritical Care Society (NCS) launched the Curing Coma Campaign, with the goal of improving the outcomes of patients with coma and DoC. The Curing Coma Campaign Scientific Advisory Council identified initial scientific challenges for the campaign [1] and partnered with the National Institutes of Health (NIH) to organize a symposium to bring together DoC experts from all over the world. These experts were invited on the basis of content expertise, ensuring diversity of specialty backgrounds to provide a forum for open scientific discussion and designate research targets for the NIH and the Curing Coma Campaign.

This NIH symposium was originally planned as a 2-day in-person meeting on the NIH campus. However, because of coronavirus disease 2019 (COVID-19)-related travel restrictions, it took place as a 2-day virtual meeting on September 9–10, 2020. This meeting was conceptualized as the first of several meetings to facilitate a broad discussion between stakeholders in the field, including scientific leaders, NIH representatives, industry partners, and patient advocates. The conference was structured along six domains (see Supplemental Figure 1): (1) defining endotypes/phenotypes, (2) biomarkers, (3) proof-of-concept clinical trials, (4) neuroprognostication, (5) long-term recovery, and (6) large data sets. A total of 471 participants registered for the meeting (376 practicing in North America, 40 in Europe, 23 in Central or South America, 11 in Asia, 10 in Africa, and 8 in the Middle East). Eleven industry representatives and sixteen National Institute of Neurological Disorders and Stroke representatives attended. Thirty-two percent of all registered participants were women. Engagement with the conference was high despite the online format, as demonstrated by attendance, which exceeded 250 both days. The discussion of topics was facilitated by using an online platform that supported an active discussion between presenters and participants.

The goal of this proceedings paper is to distill the essence of the presentations and discussions that occurred and to translate these into actionable research targets. For each topic, we summarize the background, main research gaps, overall goals, the panel discussion of the approach, limitations and challenges, and deliverables that were identified.

## Defining Endotypes/Phenotypes

### Background

Current prognostication models for brain injury that focus only on motor response and overt evidence of cognition provide limited ability to predict outcomes, with the best models having only 70–80% discrimination [2]. Treatments for acute brain injury based on phenotype classification are also greatly limited, with harmful, uncertain, or inconsistent outcomes in clinical trials [3–5]. Furthermore, most clinical trials have not successfully translated preclinical evidence of efficacy into successful therapies for humans. Thus, 60–85% of randomized controlled clinical trials (RCTs) conducted in the setting of acute brain injury have produced inconclusive results [3–5].

The limitations in accuracy of prognostication and efficacy of therapy point to the larger issue of human biological heterogeneity. Converging research demonstrates the limitations of phenotype-driven detection, diagnosis, classification, treatment selection, and prognosis for patients with severe brain injury and DoC. These approaches may lump biologically heterogeneous patients into a single phenotypic category. Successfully addressing the goal of restoring consciousness and promoting meaningful recovery requires precisely characterizing states of vigilance that distinguish the underlying etiology or pathophysiologic mechanisms affecting consciousness and/or responsiveness. By describing, defining, and classifying DoC in a manner that is more closely aligned with biological mechanisms, we can design more effective RCTs and increase the likelihood of discovering therapies that promote recovery from coma.

Clinical evaluations that focus on motor function and overt cognition fail to capture subtle but highly meaningful differences between patients [6]. One example of this is patients with covert cognition or cognitive motor dissociation (CMD). Covert cognitive states may provide a stronger basis for treatment and prognostication, but they require advanced imaging or neurophysiology to be identified [7–10].

Patients who share a specific mechanism or cluster of mechanisms that lead to the phenotype of coma are said to belong to an endotype. More generally, an endotype can be defined as a constellation of disease features anchored in a biological mechanism or pathway that is associated with a predictable disease trajectory and treatment response. An endotype is intrinsically more homogeneous than the larger phenotypically defined population. A wide variety of genetic and nongenetic determinants shape biological mechanisms that drive coma. Thus, multiple patients may be in a coma (the phenotype), but each might have a different disease trajectory, recovery potential, and treatment response depending on the underlying endotype. Each endotype will require a nuanced approach to diagnosis and treatment that would not be applicable on the phenotypic scale. The potential for endotypes to transform classification and treatment has been demonstrated in a number of domains, including cancer, asthma, and multiple sclerosis. Endotypes are a fundamental principle in personalized (precision) medicine because they may form the foundation for *prognostic enrichment*, in which participants are selected on the basis of the likelihood that they will experience a specific outcome, and for *predictive enrichment*, in which patients are selected on the basis of the likelihood that they will respond to an intervention [11–20].

### Main Research Gap (Current State)

Heterogenous pathological states with different underlying mechanisms may be imprecisely distinguished by the clinician. This imprecision leads to errors in diagnosis, prognostication, and treatment, yielding poor patient outcomes.

The overall goal is to increase diagnostic precision, build accurate prognostic models, predict therapeutic responsiveness, and help create proof-of-concept clinical trials by leveraging accurate prognostic and predictive enrichment. This approach will allow smaller mechanistically homogenous groupings of individuals who will benefit substantially more from specific treatments than the larger heterogeneous classification based on clinical phenotypes. The expectation is that this approach would increase the likelihood of recovery when treating patients with coma and DoC.

### Panel Discussion of the Approach

- Endotypes need to be characterized within the framework of the underlying, biological, mechanistic causes of the DoC, ranging from neural networks to biochemistry.
- Endotypes will require widely available techniques; in addition, a role exists for advanced imaging, electrophysiological, and other specialized tools, some of which have yet to be developed. The approach will include comprehensively applying existing and novel tools (e.g., imaging, high-dimensional data sets, statistical models, analytic techniques) that accurately assess functional and structural neural pathways related to consciousness and generate appropriate biological hypotheses.
- An example of the complexity of endotype characterization is the use of machine learning to analyze electroencephalography (EEG) or functional magnetic resonance imaging (fMRI) responses to diagnose CMD, which cannot be diagnosed by clinical examination alone. CMD is not an endotype per se, but it could be a feature characterizing a subgroup of patients who behaviorally are not overtly following commands. Taken together with subgroup-specific commonalities in network dysfunction, treatment response, and long-term outcomes, CMD could be a defining feature in one or several endotypes [7–10, 21–23]. Stated otherwise, CMD considered as a pure phenomenological state without connection to a specific biological mechanism would not alone constitute an endotype.
- Predictive enrichment in clinical trials: developing endotypes to identify patients who have high or low likelihood to benefit from an intervention will allow for selective sampling for a specific RCT and will increase the probability that an effective intervention to treat DoC will be discovered.

### Limitations/Challenges

- Endotypes should be axiomatic (i.e., mechanistically driven, associated with outcomes and with a treatment response): endotypes not meeting these minimum criteria are unlikely to be useful in the clinical domain and should not be the focus of research unless novel therapeutics are likely to leverage these mechanisms.
- Definitions and nomenclature: A review of the literature indicates that there is a high degree of inconsistency in the use of the terms (e.g., phenotype, subphenotype, endotype, endophenotype). We propose here an intuitive working definition of endotype that will need to be vetted, validated, and accepted. Conceptual validity: although the endotype paradigm has been explored in other medical domains and seems biologically plausible, research is needed to demonstrate their significance in patients with severe brain injury/DoC.
- Practical considerations: the group raised the issue of feasibility, relevance, and implementation of endotypes in the clinical setting and in the coma science community.
- There is a nonstatic nature of features within endotypes, in part, because they combine different biomarkers [24, 25] or biological processes into a single determinant factor (e.g., seizure activity, covert consciousness). To adjust for this dynamism when endotyping patients, researchers should implement clustering algorithms and be aware that a patient’s changing status may lead to endotype shifts [10, 21, 26–30]. For example, the patient’s condition in the intensive care unit (ICU) is rapidly evolving because of dynamic changes in the underlying biological mechanisms, and that evolution could be reflected in changes in endotype. This dynamism also, however, affords potential advantages, such as monitoring of duration of coma as an intermediate biomarker or assessing of endotypes as time-varying covariates.
- Validation: the group identified validation of endotypes, in light of the underlying biology, recovery probability, and response to intervention, as a major challenge.
- Censoring outcomes through premature withdrawal of life support is a confounder of research outcomes of treatment for consciousness disorders [31–34].

### Deliverables

There is a need to develop novel endotypes based on a mechanistic biological model of DoC that are not considered in current clinical treatment settings. These endotypes will inform therapeutic approaches based on personalized interventions for DoC to increase the rate of positive outcomes (i.e., regained consciousness, functional independence, improved patient-centered outcomes) among patients with DoC.

## Biomarkers

### Background

Biomarkers are considered any measurements, including chemical, physical, or biological (cellular, molecular), that relate to a biological system or the interaction between biological systems [35]. Currently available diagnostic biomarkers do not reliably detect preserved brain networks that may support the recovery of consciousness, and early prognostic biomarkers do not reliably predict outcomes [36, 37]. Because of these failings, prognostication is an uncertain art for clinicians treating DoC in the ICU and beyond. Yet many significant treatment decisions are made on the basis of uncertain prognoses. Incorrect prognosis may lead to premature withdrawal of life-sustaining therapy (WLST) or to the maintenance of individuals in a severely disabled state against their wishes. Indeed, many patients experiencing traumatic brain injury (TBI) die of WLST, despite only a portion of these patients demonstrating biomarkers associated with failure to recover consciousness [34, 38]. A quarter of patients with hypoxic-ischemic injury who died of WLST would have survived, and 16% might have made a functional recovery by the time of discharge, on the basis of their prognoses [39]. Early evidence has shown promise for magnetic resonance imaging (MRI) [7–9] and EEG [10, 40, 41] patterns that may identify individuals with covert consciousness (i.e., purposeful response to stimuli otherwise missed by behavioral assessments) [42, 43]. Biomarkers of covert consciousness may improve prognostic capabilities for patients with coma [10]. Similarly, biomarkers of covert cortical processing (i.e., association cortex responses to language otherwise missed by behavioral assessments) also may predict long-term functional outcomes [44]. A combination of model-based and data-driven analyses may enhance mechanistic understanding of DoC [37].

### Main Research Gap (Current State)

There is a lack of reliable and reproducible biomarkers for patients with DoC to assist in prognostication and serve as targets for treatments applied in clinical practice, as well as those tested in RCTs, to promote recovery of consciousness.

The overall goal is to develop a mechanistic approach to methodology, phenotyping, outcomes studies, and trial design that is anchored in biomarkers that serve as diagnostic, prognostic, monitoring, or descriptive measures in patients with DoC.

### Panel Discussion of the Approach

The Curing Coma Campaign Coma Science Working Group has identified five potential therapeutic approaches: pharmacological, electromagnetic, mechanical, sensory, and regenerative. Each provides unique opportunities to identify prognostic or therapeutic biomarkers. Proposed biomarkers can be split into four domains: molecular and cellular, imaging, electrophysiology, and transcranial magnetic stimulation and electroencephalography (TMS-EEG).

- Molecular and cellular biomarkers aim to detect neuronal and glial function, injury, death, recovery, and potential survival [45–48]. Molecular and cellular biomarkers include genetic and epigenetic proteins and cells/cellular functional assays indicative of cellular function, viability, and death. There are too many molecular and cellular biomarkers that have been proposed or implicated to comprehensively review them here, but likely a combination of markers, rather than a single one, will end up being relevant.
- Imaging biomarkers aim to map structural and functional elements of the brain, including large-scale networks. Fifteen to twenty percent of patients without signs of responsiveness, detected by either the Glasgow Coma Scale or Coma Recovery Scale, Revised, will demonstrate covert consciousness on fMRI [43]. Mapping of functional connectivity by using resting state MRI (e.g., characterization of default mode network connectivity) may help distinguish consciousness states [21, 49–53] but alone is not sufficient to predict recovery of consciousness [50, 54, 55] and should be studied along with other networks that contribute to consciousness. Ongoing studies are using such connectivity biomarkers to select patients for targeted therapies to promote recovery of consciousness [56–58].
- Electrophysiological biomarkers can be used to detect CMD [59]. EEG can be used at the bedside to detect command-following [10, 40, 41, 60] and covert cortical processing [44, 61] in patients who do not show behavioral evidence of purposeful responses. EEG can also be used to develop biomarkers of cellular preservation that relate to outcomes, such as features within burst suppression patterns, that allow for the identification of patients with and without a chance to recover [62–64].
- TMS-EEG biomarkers aim to objectively measure brain responses to direct cortical stimulation. This technique bypasses afferent sensory and efferent motor systems, which may be damaged in patients with DoC. TMS-EEG is based on an information theory that posits that conscious experience is at once highly integrated and differentiated, with this combination resulting in a complex system [65]. To measure these complex systems, a perturbation approach is most helpful to track the system’s response [66]. In practice, the brain is stimulated noninvasively via transcranial magnetic stimulation (TMS), and the brain’s response is measured with EEG. Recent work has proposed a “perturbational complexity index,” in which the numerical value is correlated with changes in consciousness, suggesting value as a marker of consciousness [67–69].

### Limitations/Challenges

- Ideal biomarkers for coma science should be safe (i.e., minimally invasive), feasible, practical (i.e., have a timely turnaround), and widely available. They should allow for accurate prognosis or goal-directed therapy and have therapeutic implications for either disease process or pathophysiology.
- Biomarkers will need to have analytical validity, clinical validity, and clinical utility established [70].
- The timing and source of sample collection, the molecules targeted, handling and processing procedures, and the end point of interest (e.g., etiology of coma, prognosis) need to be defined [71].
- Confounders in biomarker measurement that need to be considered include the impact of physiologic states and interventions on biomarkers. Targeted temperature management may affect biomarker pharmacodynamics, and sedation may affect EEG and fMRI results.
- Biases of investigators who are particularly invested in certain biomarkers need to be considered because they also may influence the goals of care decision-making processes. One of the challenges will be to temper the overinterpretation by families of results from proposed biomarker tests.
- The development of new tools must be integrated within the context of existing, albeit insufficient, biomarkers. Experimental measures need to be carefully disaggregated from proven tools in the coma research repertoire so that the accepted measures can assist in validating the new tools. It will be vital to address whether, and when, information from new tools should be shared with families, because this creates a risk of biasing the results (e.g., detection of CMD may influence the family’s WLST decision).
- Extrapolation from highly specialized centers to general practice needs to be taken into account. Coma science needs specialized care centers in which analytic tools are made widely available in a systematic, standardized way. Gaps in diagnostic, prognostic, treatment, and rehabilitation equipment and techniques extend beyond economically advantaged countries. Inequities from diagnosis to treatment and rehabilitation are a critical issue nationally and internationally.

### Deliverables

There is a need for accurate, reliable, and reproducible biomarkers for patients with DoC that allow for more precise prognostication of recovery and provide end points for RCTs.

## Proof-of-Concept Clinical Trials

### Background

Proof-of-concept clinical trials of consciousness-promoting therapies have been performed mostly in the subacute and chronic stages of DoC [72]. However, early interventions may offer opportunities to promote recovery of consciousness during critical and acute care phases of treatment. Development of early interventions for use in the ICU may prevent premature WLST, facilitate self-expression, decrease ICU-related complications, and increase access to rehabilitative care. Conversely, early-stage clinical trials may also disprove promising theoretical interventions that have not yet been fully evaluated in clinical settings. Reasons for the failure of proof-of-concept clinical trials, particularly in acute care settings, include heterogeneity of the population (including the need for better early endotyping), inadequate power, use of nonspecific assessments, timing of the intervention, and failure to account for a variety of individual mitigating factors (e.g., underlying physiology, systemic illness, comorbidities, drug interactions, timing and dosage of therapies) [73]. Additionally, the impact of the context of health care delivery and treatments may present unique challenges for clinical trials regarding psychosocial aspects of care, cultural/religious beliefs, and availability of resources throughout recovery. Over the past 2 decades, coma research has focused largely on diagnosis and prognosis to understand mechanisms of spontaneous recovery of consciousness; yet there has been little attention to mechanisms of induced recovery of consciousness. Throughout the continuum of care, coma researchers must carefully consider these aspects and design more effective clinical trials of therapies at all stages of DoC. A recent search of ClinicalTrials.gov for trials that are registered as being “in progress” used the search terms “consciousness disorder, coma, or disorders of consciousness” and found 73 trials of DoC evaluating treatment, assessment, or prognosis techniques (ClinicalTrials.gov accessed September 6, 2020), with few evaluating DoC throughout the continuum of care.

### Main Research Gap (Current State)

Patients with DoC or coma in the ICU and subacute to chronic setting are often overlooked in clinical trials, undermining the development of reliable prognostic tools and treatments.

The overall goal is to offer accurate prognostication and treatment strategies that promote recovery of consciousness throughout the continuum of care to as many patients as possible.

### Panel Discussion of the Approach

- Addressing the research gap: Researchers need to design proof-of-concept trials that focus on identifying biomarkers and neurophysiological techniques that will help diagnose, prognose, treat, and cure coma in patients. Interventional trials that aim at promoting recovery should be based on a mechanistic understanding of DoC.
- Patient selection: Both endotypes and phenotypes should be taken into account in clinical trial design. For example, individuals in minimally conscious states are more responsive to treatment than those with unresponsive wakefulness syndrome. This observation highlights the opportunities of predictive enrichment strategies to maximize clinical trial success for specific populations.
- Tested intervention: A mechanistic understanding of DoC should inform the tested intervention. One approach is use of the mesocircuit model, which allows researchers to identify areas affected by severe brain injury and the cascading effects on the system of consciousness. Depending on the affected area, different pharmacological and nonpharmacological treatments are available, including, but not limited to, amantadine [74], apomorphine, zolpidem [75, 76], TMS [77–84], transcranial direct current stimulation [85–91], deep brain stimulation [92, 93], low-intensity pulsed ultrasound, and vagal nerve or other sensory stimulation [94–96]. Specifically, within trial designs, investigators should consider and evaluate the benefits and limitations of combination therapies [72, 90]. RCTs may focus on regeneration of lost or broken circuits after TBI [77–79, 85, 86]. These techniques often seek to leverage the untapped synergistic potential of combination approaches. For example, TMS may be more efficacious when combined with a pharmacologic stimulant. Combination therapies to fix broken pathways could be defined on an individualized basis based on findings of a patient’s neurophysiologic, neuroimaging, genetic, or omics assessment (see “Defining Endotypes/Phenotypes” section for details) combined with an ever-increasing understanding of neurological pathways.
- Timing of treatment: Although early treatment may be critical to the recovery of consciousness, researchers of chronic DoC may often allow for time after the injury to decrease the confounding possibility of spontaneous recovery [77–80, 86–89, 97]. Exact timing for treatment initiation is typically determined by clinicians and researchers on an individualized basis. It is influenced by a combination of factors (e.g., past experience, patient status, time from injury). During the hyperacute and acute phases, with active inflammatory changes and secondary injury, researchers often collect data but hesitate to begin treatment to avoid causing further harm. The optimal start time, duration, and number of repetitions of an intervention needs to be investigated [73]. When defining optimal timing, the individual patient’s neurologic injury and systemic illness need to be considered.
- Biomarkers in clinical trials (see “Biomarkers” section for an overview): Clinical trials may be greatly improved by adding biomarkers, both as proxies to response and as identifiers for subpopulations (e.g., endotypes). Molecular and cellular biomarkers could serve researchers in conjunction with behavioral, electrophysiological, and imaging measures to both identify promising candidates to support recovery and track responses to investigated interventions. Although some common data elements (CDEs) may be shared with other similar disorders, many will be unique to coma. The larger the population-based group from which data are collected, the more versatile the data’s predictive capabilities. In addition, the sequence of tracking biomarkers from genome and molecules to populations is critical, and coma researchers can work from both ends: clinicians can start at the treatment population and work down toward genomic and molecular levels, whereas researchers can start at the genomic and molecular level and work up to the population of interest. This two-pronged translational and reverse-translational approach may expedite the process of identifying the best biomarkers for coma treatment and prognosis.
- Outcome measures: A mechanistic approach to clinical trial design requires new tools to identify preserved brain network connections, the ability to map the human brain networks essential for consciousness, the ability to repeatedly assess brain networks starting from the acute care phase of treatment, personalized connectome mapping tools and biomarkers, and targeted personalized treatments. Researchers agree that a multimodal approach best tracks the outcomes of patients receiving treatments. Proof-of-concept trials with small sample sizes applying physiological proxy markers (e.g., EEG enrichment) as outcome measures may allow for identification of promising treatment avenues before larger trials requiring hundreds of patients and clinical outcomes become feasible. To then evaluate effects of treatment, the primary measure should include standardized evaluation of behavioral assessment, preferably the Coma Recovery Scale, Revised, and patient-centered outcomes, such as quality of life (QOL) measures. Neuroimaging and electrophysiological scans can then be used as secondary treatment assessments.
- Improving trial design and logistics: Adaptive clinical trials, in which results can change the design of subsequent doses or trial arms, hold promise for the evolving understanding of coma. Longitudinal test–retest designs allow for alteration of drug dose, duration of therapy, dose and duration of electricity, and range of potential stimulation targets in a Bayesian-adaptive manner. Such an adaptive study was conducted among patients with COVID-19 (Randomized Embedded Multifactorial Adaptive Platform for Community-acquired Pneumonia [REMAP-CAP]) [98] and allowed for rapid development of treatments on the basis of interim analysis. The established clinical trial networks allow for efficient and rapid testing of novel interventions. This approach would be useful in a cohort of patients with coma, particularly among proof-of-concept trials that are already seeking to point the field in the correct direction rather than to serve as a final study of potential prognostic tools or treatments. The concept of pragmatism in clinical trials is crucial because it promotes later translation of trial findings into clinical practice. A role for the NCS Curing Coma Campaign to facilitate use of a master protocol could facilitate the interoperability of adaptive study data.
- Treatment standards: Establishing so-called baseline treatment standards for coma remains an important priority given current practice variations, particularly those surrounding WLST. As a criterion for enrollment in DoC and coma trials, requiring an intent to provide aggressive care and postpone WLST orders for a predetermined time frame after the injury leading to the DoC could alleviate concerns about early patient loss in acute-phase trials.

### Limitations/Challenges

Barriers for the development of strong proof-of-concept trials for curing coma science include lack of proper endotyping, lack of quality measurement tools for therapeutic response, need to account for spontaneous consciousness recovery, need for control groups, and lack of accounting for socioeconomic factors, local environment and resources, and cultural variation.

- Novelty of treatments: Essentially, all treatments currently used in patients with coma are repurposed from other pathologies and disorders. Basic and preclinical efforts should focus on identifying promising novel therapeutic approaches to cure coma. Translational scientists should help introduce and evaluate these in the clinical context of the ICU and post-acute-care setting. Close bidirectional communication between scientists who focus on clinical, translational, preclinical, and basic science aspects of coma science will be fundamental in achieving the stated goals. This bidirectional dialogue may be conceptualized in a top-down and bottom-up analogy as a translational and reverse-translational approach that needs to be central to future funding initiatives.
- Treatments may not work for all: Pharmacological treatments often work only in a small portion of the population treated [75], or they work to improve recovery only when they are being administered, and improvements disappear after treatment is stopped [74]. Nonpharmacological treatments often show limited clinical or neurophysiological effects or have highly variable effect sizes. Brain stimulation treatment trials have shown varied improvement rates depending on the treatment area, with the most promising target appearing to be the prefrontal cortex. In addition, findings from the stimulation studies suggest that individuals in minimally conscious states respond better than those in vegetative/unresponsive wakefulness states. Thus, personalized care approaches that take into account individual injuries, pathways, genetics, and omics are needed.
- Variability in trials: Variability among patients and in the diagnostic and management approaches of different clinical centers is a major challenge in trials of coma treatments. Such variability was recently highlighted by the results of the Point PRevalence In Neurocritical CarE (PRINCE) study, an observational prospective investigation of the practice of neurocritical care, which included patients with coma [99, 100]. This variability may result from equipment or staffing availability or differences in comfort with procedures across clinical centers. Consensus about endotyping of patients will be required to minimize patient variability in clinical trials. On the other hand, it may be detrimental to force clinical centers to follow overly rigid and detailed protocols because this approach may lead to the failure of a treatment at one site that succeeded at another. Additionally, variability in research infrastructure and health care service delivery infrastructure that does not support investigations of treatments/prognosis throughout the continuum of care and across treatment settings is of concern. For example, not all health centers have on-site inpatient or outpatient brain injury rehabilitation units within their health system. Patients may be enrolled into a clinical trial in the critical care or acute care setting but then be transferred to rehabilitation settings outside health systems or to independent facilities that may not have the research infrastructure to support continued participation in a clinical trial. Local experts should be able to use the techniques with which they are most comfortable, but arrangements do need to be made so that outcomes will be comparable between centers.

### Deliverables

A key deliverable is to develop trials that define biomarkers, end points, and feasible treatment approaches that build on clearly defined patient-based phenotypes and endotypes. There is a need for the design of adaptive, exploratory interventional trials that inform development of larger pragmatic trials to identify interventions that support recovery of consciousness throughout the continuum of care and improve patient-centered outcomes. Simple master protocols could facilitate development and use of these deliverables. Once effective interventions are identified, further work by using an implementation science approach will be needed to integrate findings into routine clinical care.

## Prognostication

### Background

Prognostication herewith refers to the ability of clinicians to provide surrogate decision-makers with an outlook regarding a patient’s future: the potential for recovery, the likelihood for disability, and the prospects for awakening from coma. Prognostication requires a synthesis of both data and clinical experience to inform critical decisions by surrogate decision-makers [101]. To this end, improving the accuracy of prognostication is an important aim in research focused on descriptions of clinical predictors, characterization of imaging findings, and the development of blood-based biomarkers [102].

Prognostication seeks to accurately predict whether a patient will awaken and whether they will achieve a satisfactory QOL consistent with their values and preferences. To achieve this, investigators must speak the same language as defined by CDEs. For example, “awakening” or “following commands” may be defined in different ways, as emphasized by recent work highlighting the CMD detected by EEG [10], fMRI [9], or multidimensional clinical assessments [9, 103]. Efforts to define CDEs specific to DoC are underway [1].

Yet improvements in prognostic precision are often hindered by medical decisions regarding patient triage, care limitations, or WLST. Decisions for WLST limit the unbiased study of the natural trajectories of recovery and the ability to compare these trajectories to those of patients who ultimately recover from coma [38, 39, 104]. WLST decisions after cardiac arrest are made within 72 h in as many as 63% of patients, despite findings that nearly one third of patients experience delayed awakening at a median of 93 h following arrest [39, 105]. The implications for this are grave: after cardiac arrest, early WLST results in an estimated excess of 2300 deaths in the United States each year; nearly two thirds of these patients might have had functional recovery if allowed to survive [39]. Yet significant variability exists in the rates of WLST worldwide [100]. After severe TBI, the rates of WLST vary broadly across centers [34], and extraneous factors unrelated to the medical conditions of patients may even play a role in these decisions, including geographic region, race, payment status, and other factors [106]. Ultimately, WLST decisions may result in a self-fulfilling prophecy in which false certainty about an outcome leads independently to an increase in the probability of that outcome [107].

Typically, prognostic considerations center on mortality and functional disability. However, satisfactory outcome after awakening from coma should ideally hinge on QOL, which is based on the individual patient’s values and preferences [108]. Existing research uses common functional outcome scales, such as the modified Rankin Scale or the Glasgow Outcome Scale, which focus on a limited set of disabilities, such as difficulties in walking or performing activities of daily living. These, by their nature, do not capture QOL, nor do they account for the preferences or values of patients or their families.

Recent work has highlighted cognitive or mental health disabilities that develop after critical illness, termed the “post-intensive-care syndrome” [109, 110], which may impact QOL. However, the majority of studies focused on cognitive or mental health outcome end points after critical illness have excluded patients with brain injuries [111]. Up to 20% of patients requiring care in a neuro-ICU subsequently exhibit cognitive impairment, and more than one third have at least moderate problems with anxiety or depression [112]. Those with significant cognitive or physical disability cannot be evaluated by using traditional neuropsychological testing. In a study of patient-reported outcomes after stroke, only 11.5% of those able to participate had a modified Rankin Scale score of 3 or greater [113]. The combined impacts of brain injury and critical illness on multidimensional outcomes, including physical functioning, cognition, and mental health, after recovery from coma have not been adequately studied.

### Main Research Gap (Current State)

Coma prognostication struggles to define numerous important parameters. Major gaps include defining the end points, ascertaining and accounting for WLST in coma research, defining meaningful outcomes for patients and clinicians, and establishing the validity and timing for implementation of prognostic tools. In addition, current prediction models contain high levels of uncertainty and imprecision due to a lack of complexity and multiple sources of bias. How to properly and effectively formulate and communicate prognostic judgments to families is also unknown. Physicians view prognostication as one of the most difficult parts of their profession [114, 115]; however, they receive little to no training in it, and no guidelines for how to communicate prognosis have been developed.

The overall goal is to develop tools that allow for accurate prognostication of well-defined, relevant end points for patients with DoC and to better understand the optimal methods for communicating prognosis to surrogate decision-makers.

### Panel Discussion of the Approach

- Standardization: There is a clear need to develop CDEs for established or promising diagnostic evaluations (both clinical and ancillary) and to define the optimal time window for these evaluations. There is a need to create or refine diagnostic modalities that add the greatest prognostic value at the lowest possible cost. No single test will have sufficient prognostic power to stand alone in predicting recovery from coma [116]. Therefore, many candidate tests and data will need to be evaluated simultaneously, with an aim toward multivariate model development.
- Accuracy: It is critical to reduce the uncertainty and imprecision of existing prediction models. In a study of outcome after intracerebral hemorrhage, nurses and attending physicians predicted 3-month patient outcome more reliably than the validated intracerebral hemorrhage score or predictors of functional outcome (e.g., Functional Outcome in Patients With Primary Intracerebral Hemorrhage (FUNC﻿) Score) [117]. This difference in predictive accuracy may be due to a number of factors that models do not capture, including preexisting conditions, post acute care, and patient support systems. Models developed from clinical trial data are further biased by rigid exclusionary criteria, limiting generalizability across a heterogeneous population experiencing DoC. This is a particular issue for machine learning or other artificial intelligence approaches because algorithms created with biased data will produce biased results [118].
- Validation: Prognostic tools need to be developed by using a sufficient volume of clinical information to ensure statistically robust models with adequate discrimination. Accomplishing this requires a comprehensive set of clinical data from both the acute- and post-acute-care environments across multiple centers. Coma science should practically focus on identifying predictors that incrementally enhance the prognostic accuracy of validated models.
- Calibration: To overcome uncertainty within these models, prognostic tools require careful calibration [119], a measure of how closely observed outcome occurs in relation to model predictions. A systematic review of prediction models for outcome following TBI highlighted the substantial variability in reported measures of calibration, with nearly half of all models lacking calibration statistics [119]. Clinicians should be careful to avoid models for which calibration is not known, and coma science research will require transparency in both model validation and calibration.
- Versioning: As the clinical field of coma science evolves, models may not account for updated treatment approaches (e.g., targeted temperature management) and subsequently may generate inaccurate or unreliable predictions. In this context, approaches should consider models that are adaptive to new data in real time and can continuously update their prognostic modeling [120].
- Patient-centered outcomes: Coma research will benefit from moving away from using dichotomized outcomes as an end point [121]. Instead, future studies should examine more granular outcomes focused on measures that are important to patients and based ideally on each individual’s values and preferences. Patients’ perspectives are known to change before and after disability, resulting in a “disability paradox” [122]. Despite a high rate of functional disability, most patients treated with craniotomy for ischemic stroke reported being satisfied with life [123].
- Demographic factors, such as age and sex, need to be taken into account for prognostic modeling.
- Comorbidities: Medical comorbidities and their severity are critical determinants of patient outcome and should be taken into account in prognostic tools [124–126]. Premorbid cognition, mental health, and personality are under-ascertained yet may similarly contribute to patient-centered outcomes [127, 128]. Coma science research will benefit from refining methods to comprehensively evaluate the patient’s comorbid status.
- Early WLST: Methods to reliably ascertain and statistically address WLST in patients with DoC are important. Efforts to study outcomes in populations with high mortality [129] should include considerations for when death results from medical decision-making. Additional considerations include protocolized approaches to limit inappropriately early WLST within the context of clinical study design to avoid confounding results or designing studies for populations within cultures or societies that do not practice WLST to the same degree.
- Communicating outcome: Clinicians face a schism between their implicit conceptualization of good outcome and the definitions brought by the patients and their families [108]. Clinicians may bring their personal biases to their perspective on what is considered a good outcome for patients [130, 131]. To avoid this bias, clinicians and researchers must increase their own awareness of the discordance between what clinicians think families should receive in terms of information and support and what families actual need [114]. Numerous factors may contribute to this discordance in communication and decision-making [114, 132], and the existing gaps in communication cannot be filled until clinicians have a better understanding of current prognostic communication practices. Clinicians should encourage families and surrogates to express a patient’s own values and preferences and then modify their prognostic communication on the basis of these [133].

### Limitations/Challenges

- Population-based data: Current prognostic tools are based on large aggregated patient cohorts and not on individualized measures. Hence, outcome assessments historically have lacked consideration of patient-specific outcomes of interest. Although population-based data are needed for statistical power, unmet challenges include curating comprehensive clinical data anchored by CDEs and a better understanding of biologically relevant and patient-centered end points.
- Bias: Unmeasured bias in clinician to decision-maker relationships and communication may confound predictions. As new prognostic tools are developed, researchers need to avoid introducing personal or data biases during the design process. A critical source of bias that touches both limitations may occur during communication between clinicians and patient surrogates. New tools are needed to harmonize prognostic communication to ensure that patients’ cultural and social desires are considered during assessment and treatment decision-making [133, 134]. Prognostic tools are meant to serve not only clinicians but also families, who must make decisions about the continued care of their loved ones [133]. One qualitative study of patients with TBI found that patient surrogates preferred numeric prognostic probabilities, whereas physicians tended to provide qualitative prognosis, in part, because of their underestimation of families’ ability to understand them [114]. Adding to this complexity are unspoken frustrations surrogates feel about uncertainty and unintentional perceptions of certainty created by physicians [114]. For patients with DoC, this is particularly important, and the best methods to reduce the bias introduced by how prognostic communication is approached are currently unknown.
- Communication: On the basis of a policy statement from leading critical care societies, communication with surrogates and families should not be limited to a single meeting [133]. Instead, clinicians should build trust and form relationships through frequent contact with families, helping them understand the evolving condition of the patient and discussing both treatment options and potential outcomes [133, 135]. Meeting regularly with families may be difficult for clinicians who are already overburdened in their tasks and overstretched in their time. However, this open communication is recommended by critical care societies and the Institute of Medicine [133, 136]. The clinician–family relationship should include the option for families to obtain second opinions from unbiased clinicians. Another key element of open communication, commonly requested by families, is clinician humility in admitting what information is uncertain about a patient’s prognosis or treatment [114, 137]. Finally, clinicians should strive to be open with families—not only about treatment but also about any issues (such as cultural differences, past experiences, racism, and more) that may complicate communication itself [138].
- Nonmedical factors in clinical care: Clinicians are not ideally equipped to determine nonmedical aspects of prognosis, which include social support, spiritual beliefs, and socioeconomic factors. An interprofessional approach (including social workers and pastoral care) has been recognized as a key component in providing high-quality critical care to complex and diverse patients [139–141]. Institutional support for these aspects of care are often limited, with care burden falling to patients and their families. The process of organ transplantation includes a framework for considering the nonmedical aspects of care. [142]. The evaluation of psychosocial support structures available to transplant patients has been well established. By contrast, there is little understanding of the structures of support for patients with acute brain injury [143].
- Nonmedical factors in coma science: coma science similarly should seek to better quantify social support, spiritual and religious influences, personal priorities, social and cultural values, and socioeconomic status when considering prognosis.

### Deliverables

There is a need to develop (1) accurate, reliable, and reproducible prognostic tools for patients with DoC and (2) empirically grounded interventions for high-quality prognostic communication that promotes shared decision-making between clinicians and decision-makers.

## Long-Term Recovery

### Background

Survival of patients with coma who previously would have died is now common because of improvements in early resuscitation, interventions, and ICU management. TBI outcomes have improved through advances in prehospital triage, rehabilitation care, and compliance with published clinical treatment guidelines [144, 145]. In addition, survivors of cardiac arrest with hypoxic-ischemic brain injury are a new population for neurorehabilitation specialists to treat because of increased survival secondary to mainstream use of therapeutic hypothermia and through public access defibrillation efforts and compression-only resuscitation education [146, 147]. Evidence cited in the 2018 American Academy of Neurology, American Congress of Rehabilitation Medicine, and National Institute on Disability, Independent Living, and Rehabilitation Research practice guideline update on DoC makes clear that recovery from coma continues longer than previously believed, leading to meaningful functional improvement in a substantial minority of those affected [148]. The potential for good recovery in those with DoC supports the overarching goal of prospectively studying long-term recovery trajectories for this population.

Acute treatment of coma and other DoC appropriately focuses on short-term survival and recovery of gross neurological function but often fails to consider changes that occur during the post acute course, ultimately influencing long-term outcome. This can result in underestimation of prognosis and inappropriate treatment decisions. In the 1994 *New England Journal of Medicine* report on the persistent vegetative state, outcome only extended to 1 year after treatment [149]. A few facilities have developed specialized programs for patients with DoC, but admissions are constrained by fiscally driven gatekeeping policies, providing limited opportunity for systematic outcomes research on this population. Patients without detectable signs of recovery at the time of acute care discharge may be thought to be destined for unfavorable recovery and, thus, may not be referred to or accepted into rehabilitation centers. Many are routed to nursing facilities and home care settings that are ill-equipped to manage the complex medical and neurobehavioral consequences of the injury.

There is a need for evidence-based post acute care that spans a variety of settings. A study of early functional outcomes of 396 patients with traumatic DoC and without the ability to follow commands who were admitted to inpatient rehabilitation showed that 68% of patients were able to follow commands, 23% of patients had recovered from posttraumatic amnesia, and 7–14% of patients were independent on a range of self-care and mobility tasks (dependent on skill area assessed) prior to rehabilitation discharge. There is also an imbalance in funded research for patients with traumatic versus nontraumatic injuries, leading to a dearth of data regarding recovery in patients with nontraumatic DoC.

Active medical management by clinicians with expertise in brain injury reduces the rate of new complications in patients with DoC [150]. Acute care physiatry input can help initiate early DoC prognostication efforts, prevent complications, support early efforts to improve level of consciousness, and promote safe transitions of care [151].

Research conducted on long-term functional outcome after TBI through the TBI Model System Program found that by 5 years post injury, 74% of patients who were admitted to inpatient rehabilitation and unable to follow commands had regained this ability, approximately 20% were able to live without in-home supervision, and 19% were rated as capable of competitive employment [152]. Measurable gains in independence continued out to 10 years in a portion of the same cohort [153], particularly those who recovered command-following during their rehabilitation stay. The recovery trajectory for those with nontraumatic DoC differs but is less well understood. Pooled analysis of patients with prolonged nontraumatic vegetative state/unresponsive wakefulness syndrome suggests that 17% will recover consciousness at 6 months and an additional 7.5% beyond 6 months [154].

These findings indicate that there is substantial recovery over a long period of time for a sizable minority of individuals with prolonged DoC. Although surviving patients often have a high comorbidity burden, effectively managing comorbidity appears to improve long-term outcome [150, 151]. Emerging treatments in the field may enhance these positive long-term outcomes further [72]. This potential for long-term recovery needs to be considered when delivering acute care early after the injury as well as when triaging patients into the ideal care setting to support long-term recovery.

### Main Research Gap (Current State)

Although understanding of the long-term recovery trajectory of patients with DoC has expanded considerably over the last decade, predicting the recovery trajectory and functional outcome remains imprecise in individual patients, particularly in the very early period when many urgent treatment decisions are being made.

### Overall Goal

Long-term, researchers need to characterize the clinical trajectory of a broad range of DoC patients over a long time frame, taking patient heterogeneity and contextual factors into account. As a short-term goal, studying those with good and poor recovery after DoC may be a critical first step to help demonstrate and define the biomarkers, early treatments, and personal or environmental factors that represent, enhance, and discern potential for favorable outcome.

### Panel Discussion of the Approach

To be successful, longitudinal outcome research involving patients with DoC must address the following considerations:

- Combating pessimism: A challenge of long-term recovery is promoting optimism in the clinical team responsible for caring for the patient. The neurocritical care team should partner with other disciplines, including neurorehabilitation specialists and social workers. These partnerships extend the breadth of knowledge necessary to ensure accurate diagnosis, improve outcome prediction and prognostic counseling, identify short- and long-term care needs, and establish comprehensive treatment regimens. This approach fosters a more thorough understanding of the patient’s condition and the probability of further recovery, which can boost the optimism of the neurocritical care team and sustain high engagement in care.
- Longitudinal outcome data: Investigations of long-term outcome following severe brain injury have historically employed 6- or 12-month study end points. In 1994, the authors of a major review [149, 155] were able to find outcome data later than 12 months in less than 50 patients with TBI. The challenges associated with obtaining long-term outcome data from patients with DoC make it difficult to build large data sets necessary for tracking the natural history of recovery. Point-of-care or other carefully designed longitudinal studies are needed to acquire long-term data, and study end points should exceed 12 months because there is growing evidence that meaningful recovery can continue for at least 10 years [153].
- Conducting pragmatic clinical trials that investigate the context of care delivery (i.e., inpatient rehabilitation facility vs. home vs. nursing home facility) on long-term recovery of patients with DoC.
- Large multicenter clinical trials: The field needs large RCTs to assess any of the proposed mechanisms of treatment for both short- and long-term outcomes. However, large trials alone will not solve a number of structural data insufficiencies, such as survivor bias, diversity of injury etiologies, and the many aspects of social determinants of health. These can be addressed by using strategies that may include oversampling for underrepresented groups, provision of rehabilitation for all enrolled, and standardized acute- and post-acute-care treatment models. Recruiting large samples of patients with DoC will require a network of sites for recruitment, especially to ensure a large enough population to apply endotypes.
- Ethical questions of equity in clinical trial participation of patients with DoC, autonomy of decision-making, and the implications of CMD need to be discussed, particularly if long-term care is required.
- Impact of cultural perspectives on neurorecovery and disability: The DoC patient population is culturally diverse, which means that patients and surrogates have distinct sets of values, customs, and cultures and a variety of perspectives on health, wellness, life, and disability. These beliefs impact multiple aspects of care, including access to services, treatment opportunities, community engagement, and QOL. This work may require the assistance of a cultural representative who can support the conversation by translating a family’s needs to a clinician.

### Limitations/Challenges

Research involving patients with DoC is hampered by multiple factors, including the following:

- Early WLST in patients who would otherwise go on to attain significant recovery of function.
- Discriminatory payer policies limit access to specialized post-acute-care facilities that have been shown to reduce complications in patients with DoC.
- Variability in clinician knowledge and approach to communicating prognostic information may negatively impact caregiver understanding, further increasing the probability of early withdrawal of aggressive treatment.
- Difficulty integrating data from different systems of care that are not comparable and do not permit data sharing.
- Ethical implications of clinical trial participation for long-term recovery of DoC.
- Limited and regionally variable psychosocial support for caregivers following discharge from the acute care setting. Some consumer-created, well-organized caregiver support and advocacy groups do exist; however, most resources are available for patients with TBI rather than for those with non-TBI. The impact of advocacy groups in promoting access to care has not been formally studied and remains unknown.

### Deliverables

Deliverables include (1) determining the natural history of recovery from DoC across the lifetime, (2) identifying accurate biomarkers and clinical predictors of favorable and unfavorable outcome, and (3) understanding the effectiveness of specific interventions on DoC recovery trajectory.

## Large Data Sets

### Background

Large data sets, or databases, can help address multifaceted challenges of DoC research, which includes medical, scientific, technical, ethical, and social dimensions. Large data sets will help coma researchers by (1) fostering international consensus on issues such as definitions, causes, and confounders of DoC through implementation of CDEs [156]; (2) facilitating the design of operative diagnostic tools and meaningful outcome time points [157]; (3) enabling better research, including epidemiological studies that take advantage of between-center differences that explore processes of care and structural/organizational factors that may impact outcome and treatment heterogeneity effects [158–160]; (4) organizing and federating an international network of coma scientists who share a common language and connection [100]; (5) improving patient care by standardizing results and improving expertise [161]; and (6) helping family members and caregivers of patients with coma provide better decision-making tools based on improved knowledge of acute- and post-acute-care trajectories [162]. A survey of the Scientific Advisory Council of the Curing Coma Campaign and the broader NCS found that clinicians and investigators’ interests focus on research involving patients affected by coma and acute DoC. These individuals generally have access to clinical data but lack access to other data types (e.g., anatomical data, clinical outcomes, safety).

The panelists surveyed current data sets to determine whether existing resources could be expanded or absorbed into a large international data set (see Table 1). Currently available resources are almost exclusively limited to patients with TBI or cardiac arrest. Available limited international data are disease specific (e.g., TBI, seizures), with relatively small numbers of patients with coma; moreover, these data pay little attention to the origins of DoC (e.g., toxic metabolic).

**Table 1:** Large data sets detailed study information

Trial/database	University/sponsor	Description	Population	Sample size	Patients with coma	MRI	EEG	Behavioral assessment	Outcome scale	Outcome time points
TRACK-TBI [167, 169]	University of California, San Francisco; NINDS/private	Multicenter observational	TBI	3211	150–200	Yes (2 weeks, 6 months)	No	CRS-R	GOS-E, DRS	2 weeks; 3, 6, and 12 months; 7 years
CENTER TBI [170, 171]	European Union	Multicenter observational	TBI	4509 (47% ICU)	968	Yes (subset)	No	GCS, CRS-R	GOS-E	6 months (< 5% 2 years)
ProReTro Database [172]	French Ministry of Health, France	Multicenter observational	Acute brain injury	310	310	Yes (subset)	Yes (*n* = 270)	GCS	GOS, mini mental state	1 months
Databank MRI-COMA [173, 174]	Assistance Publique–Hôpitaux de Paris, France	Multicenter observational	CA, ICH, SAH, TBI	218	218	Yes	No	Not answering simple orders	GOS-E	1 years
RECONFIG [175]	Columbia University, NINDS	Multicenter observational	ICH	120	120	Yes (acute)	Yes	CRS-R	GOS-E, mRS, neuro-QoL, TICS	6 months, 3 and 5 years
CONSCIOUSNESS [10]	Columbia University	Single-center observational	Acute brain injury	150	150	Yes (subset acute)	Yes	CRS-R	GOS-E, mRS	3, 6, and 12 months; 3 and 5 years
RESPONSE [176]	Massachusetts General Hospital	Single-center observational	TBI	75	75	Yes (acute, 6 months, 1 and 3 years)	Yes (acute, 6 months)	GCS, CRS-R	GOS-E Revised, DRS, BTACT	6 months; 1, 3, and 5 years
SAHIT [177]	Canadian Institutes for Health Research	Mixed (9 RCTs, 5 cohort studies)	SAH	11,443	1250	No	No	No	GOS-E, mRS	1 years
TED [6, 178]	University of California, San Francisco, US Department of Defense	8 studies	TBI	6814	Subset	Yes (subset)	No	CRS-R (TRACK-TBI only)	GOS-E, DRS	1 years

*BTACT* Brief Test of Adult Cognition by Telephone, *CA* cardiac arrest, *CENTER TBI* Collaborative European Neurotrauma Effectiveness Research in Traumatic Brain Injury, *CONSCIOUSNESS* Consciousness Recovery Project with Outcomes, *CRS-R* Coma Recovery Scale revised, *DRS* Disability Rating Scale, *EEG* electroencephalography, *GCS* Glasgow Coma Scale, *GOS-E* Glasgow Outcome Scale Extended, *ICH* intracerebral hemorrhage, *ICU* intensive care unit, *MRI* magnetic resonance imaging, *MRI-COMA* Multimodal Resonance Imaging for Outcome Prediction on Coma Patients, *mRS* modified Rankin Scale, *neuro-QoL* quality of life in neurological disorders, *NINDS* National Institute of Neurological Disorders and Stroke, *ProReTro *Prognosis of Brain Reflexes, *RCT* randomized controlled clinical trial, *RECONFIG* Recovery of Consciousness Following Intracerebral Hemorrhage, *RESPONSE* Resting and stimulus-based paradigms to detect organized networks and predict emergence of consciousness, *SAH* subarachnoid hemorrhage, *SAHIT* Subarachnoid Hemorrhage International Trialists, *TBI* traumatic brain injury, *TED* TBI Endpoints Development, *TICS* Telephone Interview for Cognitive Status, *TRACK-TBI* Transforming Research and Clinical Knowledge in Traumatic Brain Injury

### Main Research Gap

Limited large data sets of patients with DoC exist at this time. Gaps in available data sets include limited imaging and electrophysiologic data, lack of CDEs tailored to DoC, focus on primary disease states (i.e., TBI, intracerebral hemorrhage, etc.) rather than coma, lack of applicable biomarkers for coma and DoC, fragmented acute and chronic care data, and regional data restricted primarily to the United States and Europe. The key challenges to creating a large coma science data set are lack of uniform global data standards and collection across both substantive measures (i.e., what is collected) and data handling and storage mode.

The overall goal is to gain insights into mechanisms, predictors, and trajectory of recovery and lay the foundation for interventional trials by creating a large international data set of patients with DoC.

### Panel Discussion of the Approach

- To accomplish this goal, researchers must develop a database that incorporates the following: a high-throughput neuroinformatics platform [163] with real-time clinical and research input, automated imaging segmentation and analytical tools for quantification [164], remote computation and Web-based user interfaces, a biobank, long-term follow-up data, data sharing agreements, and an international open access governance structure.
- Such a database would seek to capture data that cover the trajectory of the disorder. This scope may require enrolling healthy populations to track patients who experience DoC from initial onset to death in a longitudinal model similar to the Framingham Heart Study [165].
- The database would collect clinically meaningful data at standardized intervals in real time and provide space for raw data (e.g., neuroimaging, neurophysiology, serological, pathological, multimodal). The associated biobank would collect clinically meaningful samples at standardized intervals using standardized practices (i.e., sampling, handling, processing) for delivery to a centralized repository with disaster-proof storage. The biobank may include a brain bank for whole-brain specimens.
- The database platform should be automated for real-time data input into a structured data architecture. Automation requires no manual data entry; instead, a tool would retrieve information from standardized electronic health records. The database should require only periodic human maintenance, with manual validation of archived data to ensure reliability. Statistical control processes existing in other industries would be used to check data quality for completeness and consistency.
- Its governance model, which would aim to promote equal stakeholder access, would include a central oversight board and incorporate FAIR (Findable, Accessible, Interoperable, Reusable) and open science principles. Any legal liability for using the database would lie with the end user, but oversight would discourage unethical use.
- The database should be accessible through an open access, secure Internet-based interface that would be intuitive and user-friendly. The output should be flexible to allow for project-specific programming by the end user. In cases in which project-specific programs may prove useful to the community at large, the platform may also incorporate new technologies developed by individual users. A federated approach for data archival, analysis, and result sharing would be ideal.
- Initial steps: The Curing Coma Campaign is not currently prepared to create such a complex system without a robust foundational framework. (1) To start, a deep and broad survey of the current database landscape is needed to provide a better understanding of what resources are available and which require creation. (2) In parallel, CDEs must be defined. (3) Once language, resources, wants, and needs are established, a simple database to obtain data from patients with coma can be built that begins standardizing data input and structure, types of patients and disorders covered, and current diagnostic and management strategies. (4) Once the initial database is established, existing frameworks could guide its expansion (e.g., the Alzheimer’s Disease Neuroimaging Initiative [166] and Transforming Research and Clinical Knowledge in Traumatic Brain Injury [167]) to include increasingly complex data and automated data input. (5) The final result will be a comprehensive international repository for research on coma and DoC. This ultimate version of the database must balance comprehensiveness and flexibility to incorporate future progress as the field advances its understanding.

### Limitations/Challenges

There are several major challenges for creating an international data set:

- The integration of data from different countries [168] by using a variety of data collection platforms (i.e., electronic medical vs. traditional paper records) [99].
- Data elements need to be harmonized.
- Long-term data capture will require infrastructures that may otherwise not be available globally.
- The Curing Coma Campaign will need to create guidance for updating and upgrading both the technology and the clinical practices of global sites treating patients with coma.
- Existing databases do not specifically address DoC or coma and will require careful data mining but may provide a launching point for the larger database. Thus, another approach is to start to collate existing neurological research databases.
- Balancing simplicity to allow for global access and data entry, with complexity and depth of data required to address scientific questions: researchers will need to leverage innovative technology to create a self-maintained system that can retrieve data from electronic medical records and laboratory reports and make it accessible and available globally.
- Lack of data sharing and master trial agreements may require Curing Coma Campaign researchers to assist sites with their creation to match the new technological requirements.
- Obtaining funding for this enterprise will require government support and global lobbying.

### Deliverables

Deliverables include (1) creation of data dictionaries based on CDEs, common language and terminology, and data structure framework and (2) a large simple international database on patients with DoC that is built on CDEs.

## Overall Conclusion

The level of engagement during the first NIH symposium of the Curing Coma Campaign supports the conclusion that this was a major success. The gaps, goals, approaches, and deliverables outlined above are a direct result of the symposium and provide an actionable path for the campaign toward achieving its overall goal of curing coma. Research efforts will require close communication and coordination between those focusing on clinical, translational, preclinical, and basic science aspects of coma science to make great advances and cure coma. Future meetings are planned and will focus on additional areas of high importance for the Curing Coma Campaign, such as ethical implications of DoC research and the development of CDEs.

## Supplementary Information

Below is the link to the electronic supplementary material.

Supplementary file1 (DOCX 34 kb)

Supplementary file2 (PDF 581 kb)
