# Supplementary material for: Proceedings of the First Curing Coma Campaign NIH Symposium: Challenging the Future of Research for Coma and Disorders of Consciousness
Source: Neurocrit Care. 2021 Jul 8;35(Suppl 1):4–23. doi: 10.1007/s12028-021-01260-x (PMC8264966; doi:10.1007/s12028-021-01260-x)
Supplement: Supplementary file 1 — Supplementary file1 (DOCX 34 kb) [file 12028_2021_1260_MOESM1_ESM.docx]

| Nigatu | Abate | abate.nigatu@gmail.com | Ethiopia |  |
| --- | --- | --- | --- | --- |
| Yasser B. | Abulhasan | yasser.abulhasan@hsc.edu.kw | Kuwait |  |
| M Sohel | Ahmed | drsohel4nicu@yahoo.com | United States of America | |
| Venkatesh | Aiyagari | venkatesh.aiyagari@utsouthwestern.edu | United States of America | |
| Yousaf | Ajam | yoajam@utmb.edu | United States of America | |
| Yama | Akbari | yakbari@uci.edu | United States of America | |
| Raid | Alatassi | atassi73@hotmail.com | United Kingdom |  |
| Asher | Albertson | albertsona@wustl.edu | United States of America | |
| Sheila | Alexander | salexand@pitt.edu | United States of America | |
| Ayham | Alkhachroum | albertsona@wustl.edu | United States of America | |
| Sharon | Allen | axa2610@med.miami.edu | United States of America | |
| Nawaf | Almeshal | nawaf.almeshal@jhsmiami.org | United States of America | |
| Sulaiman | Almohaish | almohaishsm@vcu.edu | United States of America | |
| Fawaz | Al-Mufti | fawazalmufti@outlook.com | United States of America | |
| Yazeed | Alolayan | dr.yazeed.ola@gmail.com | United States of America | |
| Ali | Alsanousi | ali.alsanousi@me.com | Kuwait |  |
| Diana | Alsbrook | dalsbro2@uthsc.edu | United States of America | |
| Ronald | Alvarado | Ronald.alvaradodyer@uchospitals.edu | United States of America | |
| Pouya | Ameli | pouya@ufl.edu | United States of America | |
| Ushtar | Amin | Uamin@usf.edu | United States of America | |
| Edilberto | Amorim | amorim@ucsf.edu | United States of America | |
| Mohammad | Anadani | dr.anadani@gmail.com | United States of America | |
| Jitka | Annen | jitka.annen@uliege.be | Belgium |  |
| Salah | Aoun | salah.aoun@utsouthwestern.edu | United States of America | |
| Brian | Appavu | bappavu@phoenixchildrens.com | United States of America | |
| Nayelli | Argüelles Morales | n_arguelles@yahoo.com | Mexico |  |
| Tashan | Arrivas | tashanh@msn.com | United States of America | |
| Yegeta | Asfaw | Yegeta16@gmail.com | Ethiopia |  |
| Alyssa | Avilez | avileza@uci.edu | United States of America | |
| Rafael | Badenes | rafaelbadenes@gmail.com | Spain |  |
| Mary Kay | Bader | Badermk@aol.com | United States of America | |
| Neeraj | Badjatia | nbadjatia@umm.edu | United States of America | |
| ROUNAK | BAGHBANINOGOURANI | BAGHBANR@UCI.EDU | United States of America | |
| Clotilde | Balucani | cbaluca1@jh.edu | United States of America | |
| Alice | Barra | mbarra@mgh.harvard.edu | Belgium |  |
| Megan | Barra | a.barra@uliege.be | United States of America | |
| Ximena | Barrera | Chr481llc@gmail.com | United States of America | |
| DORMAR DAVID | BARRIOS MARTINEZ | dormar.barrios@fsfb.org.co | Colombia |  |
| James | Bartscher | jfbartscher@gmail.com | United States of America | |
| Rachel | Beekman | Rachel.Beekman@yale.edu | United States of America | |
| Kathleen | Bell | Kathleen.Bell@UTSouthwestern.edu | United States of America | |
| Sarah | Bell | sarah.bell@nih.gov | United States of America | |
| Patrick | Bellgowan | psfb@mail.nih.gov | United States of America | |
| Francis | Bernard | bernard.francis@gmail.com | Canada |  |
| James | Bernat | bernat@dartmouth.edu | United States of America | |
| Ritwik | Bhatia | Ritwikbhatia89@gmail.com | United States of America | |
| Marek | Binder | marek.binder@uj.edu.pl | Poland |  |
| Stefanie | Blain-Moraes | stefanie.blain-moraes@mcgill.ca | Canada |  |
| Thomas | Bleck | tbleck@gmail.com | United States of America | |
| Yelena | Bodien | ybodien@mgh.harvard.edu | United States of America | |
| Melanie | Boly | boly@wisc.edu | United States of America | |
| Estelle | Bonin | estelle.bonin@uliege.be | Belgium |  |
| Naomi | Booker | naomi.booker@nih.gov | United States of America | |
| Zhanna | Botta | Logo1406@yandex.ru | Russian Federation | |
| Marie-Michele | Briand | Marie-michele.briand.1@ulaval.ca | Canada |  |
| Gretchen | Brophy | gbrophy@vcu.edu | United States of America | |
| Jeremy | Brown | Jeremy.brown@nih.gov | United States of America | |
| Enzo | Brunetti | enzo.brunetti@gmail.com | Chile |  |
| Eder | Caceres | edercr@clinicaunisabana.edu.co | Colombia |  |
| Melissa | Cagnina | melissa.cagnina@va.gov | United States of America | |
| Joshua | Cain | joshcain@ucla.edu | United States of America | |
| PAMELA | CAISON | msepiphany@yahoo.com | United States of America | |
| eusebia | calvillo | ecalvil2@jhmi.edu | United States of America | |
| YESICA | CAMPOS | YESICA.CAMPOS@UTSouthwestern.edu | United States of America | |
| Raphael | Carandang | raphael.carandang@umassmemorial.org | United States of America | |
| Paolo | Cardone | paolo.cardone95@gmail.com | Italy |  |
| Julia | Carlson | jcarlson13@mgh.harvard.edu | United States of America | |
| Kyle | Carpenter | kylecarpenter05@gmail.com | United States of America | |
| Felipe | Carvajal | quasarlux@icloud.com | Argentina |  |
| Felipe | castro | fcastro13@yahoo.com | Colombia |  |
| Ramiro | Castro-Apolo | drgcastro690@gmail.com | United States of America | |
| Benedetta | Cecconi | benedetta.cecconi@uliege.be | Belgium |  |
| Emilio | Cediel | 2emiliodn@gmail.com | Colombia |  |
| Cherylee | Chang | cherylee.chang@duke.edu | United States of America | |
| Lily | Chau | lily.chau@wustl.edu | United States of America | |
| Daofen | Chen | daofen.chen@nih.gov | United States of America | |
| Amy | Cheung-Taylor | amy.ctaylor@yahoo.com | United States of America | |
| Sung-Min | Cho | csmfisher@gmail.com | United States of America | |
| Sherry | Chou | chouh@upmc.edu | United States of America | |
| David | Chung | dychung@mgh.harvard.edu | United States of America | |
| Rosario | Ciliento | rosario.ciliento@gmail.com | Italy |  |
| Jan | Claassen | jc1439@cumc.columbia.edu | United States of America | |
| Liesl | Close | liesl.close@jhsmiami.org | United States of America | |
| Holly | Cohan | holly.cohan@jhsmiami.org | United States of America | |
| Beth | Crago | ecrago@pitt.edu | United States of America | |
| Claire | Creutzfeldt | clairejc@uw.edu | United States of America | |
| Bill | Curley | wcurley@mgh.harvard.edu | United States of America | |
| Daniel | Daneshvar | ddaneshvar@partners.org | United States of America | |
| Neha | Dangayach | neha.dangayach@mountsinai.org | United States of America | |
| Jeremiah | Darnell | jeremiah.darnell@integralife.com | United States of America | |
| Sabrina | De Marco | sabri.dmarco@gmail.com | Argentina |  |
| Joanne | deKay | jdekay@mmc.org | United States of America | |
| masoom | desai | dr.masoom@gmail.com | United States of America | |
| Rajat | Dhar | dharr@wustl.edu | United States of America | |
| Michele | Dillon | michele.dillon@roseliassociates.com | United States of America | |
| Michael | Diringer | diringerm@wustl.edu | United States of America | |
| Jonathan | Dissin | dissinj@einstein.edu | United States of America | |
| Kevin | Do-Tran | kdotran@uci.edu | United States of America | |
| Lily | Dourlain | ldourlain@moberg.com | United States of America | |
| Catherine | Duclos | catherine.duclos@mail.mcgill.ca | Canada |  |
| Peter | Dziedzic | phd@jhu.edu | United States of America | |
| Brian | Edlow | bedlow@mgh.harvard.edu | United States of America | |
| Ahmed | Elkady | ahkady86@gmail.com | Saudi Arabia |  |
| Ivette | Emery | emeryi@mmc.org | United States of America | |
| Nathan | Evanson | nathan.evanson@cchmc.org | United States of America | |
| Matteo | Fecchio | mfecchio@mgh.harvard.edu | United States of America | |
| Elana | Felder | efelder@binausa.org | United States of America | |
| Monica | Ferrea | mferrea@gmail.com | Argentina |  |
| Jackie | Feyereisen | Jackie.feyereisen@northmemorial.com | United States of America | |
| Joseph | Fins | jjfins@med.cornell.edu | United States of America | |
| David | Fischer | d.b.fisch@gmail.com | United States of America | |
| Eileen | Fitzpatrick DeSalme | efitzpat@einstein.edu | United States of America | |
| Brandon | Foreman | foremabo@ucmail.uc.edu | United States of America | |
| Allison | Frantz | allison.frantz@mail.mcgill.ca | Canada |  |
| Aina | Frau-Pascual | afraupascual@mgh.harvard.edu | United States of America | |
| W David | Freeman | freeman.william1@gmail.com | United States of America | |
| Tzippy | Friedler | tfriedler@binausa.org | United States of America | |
| Jennifer | Frontera | Jennifer.frontera@nyulangone.org | United States of America | |
| Katerina | Fufaeva | k.fufaeva@gmail.com | Russian Federation | |
| Nashla | Garcia | nashlag@yahoo.com | United States of America | |
| Gabriel | García | gabrielgarciam002@gmail.com | Ecuador |  |
| Abraha | Gebreegziaher | abreha_gebreegziabher@yahoo.com | Ethiopia |  |
| Meron | Gebrewold | merianair@yahoo.com | Ethiopia |  |
| Romer | Geocadin | rgeocad1@jhmi.edu | United States of America | |
| Benjamin | George | benjamin_george@urmc.rochester.edu | United States of America | |
| Joseph | Giacino | jgiacino@mgh.harvard.edu | United States of America | |
| marisa | gilbert | marisa.gilbert@jhsmiami.org | United States of America | |
| Michael | Giordano | mgiordano@wisc.edu | United States of America | |
| Rosemarie | Girardeau | Rosemarie.girardeau@integralife.com | United States of America | |
| Chavie | Glustein | cglustein@binausa.org | United States of America | |
| FERNANDO | GOLDENBERG | fgoldenb@uchicago.edu | United States of America | |
| Christian | Gonzalez | Ch.gonzalezv@gmail.com | Chile |  |
| Urszula | Gorska | gorska@wisc.edu | Poland |  |
| Olivia | Gosseries | ogosseries@uliege.be | United States of America | |
| Ebonye | Green | msgreen77@gmail.com | United States of America | |
| Theresa | Green | theresa.green@uq.edu.au | Australia |  |
| David | Greer | dgreer@bu.edu | United States of America | |
| Brooks | Gross | brooks.gross@nih.gov | United States of America | |
| Mary | Guanci | Mmguanci1@gmail.com | United States of America | |
| Cristian | Gutierrez Alvarez | cristian.gutierreza@udea.edu.co | Colombia |  |
| Daniel | Habboush | dhabboush@moberg.com | United States of America | |
| Maged | Habiba | magedhabiba@icloud.com | Egypt |  |
| Casey | Hall | clhall2@emory.edu | United States of America | |
| Shelby | Halsey | shelby.halsey@utsouthwestern.edu | United States of America | |
| Flora | Hammond | flora.hammond@rhin.com | United States of America | |
| Sangwoo | Han | sangwh1@hs.uci.edu | United States of America | |
| Daniel | Hanley | dhanley@jhmi.edu | United States of America | |
| Holly | Harrington | hharrington@infinityconferences.com | United States of America | |
| Jed | Hartings | jed.hartings@uc.edu | United States of America | |
| Adam | Hartman | adam.hartman@nih.gov | United States of America | |
| Raimund | Helbok | raimund.helbok@tirol-kliniken.at | Austria |  |
| Claude | Hemphill | claude.hemphill@ucsf.edu | United States of America | |
| Alison | Herman | ali.herman@yale.edu | United States of America | |
| Christian | Hernandez | christian.e.hernandez@duke.edu | United States of America | |
| Cynthia | Hill | cynthia.hill@utsouthwestern.edu | United States of America | |
| Michelle | Hill | michellehillcns@gmail.com | United States of America | |
| Archana | Hinduja | archana.hinduja@osumc.edu | United States of America | |
| H.E. | Hinson | hinson@ohsu.edu | United States of America | |
| Karen | Hirsch | khirsch@stanford.edu | United States of America | |
| Mohammad | Hirzallah | mhmd.hirz@gmail.com | United States of America | |
| Mary Ellen | Hook | maryellen.hook@bryanhealth.org | United States of America | |
| Jonatan | Hornik | jonyhornik@gmail.com | United States of America | |
| Xiao | Hu | Xiao.hu@duke.edu | United States of America | |
| Theresa | Human | theresa.human@bjc.org | United States of America | |
| Rashid | Hussain | Rashid.S.Hussain@gmail.com | United States of America | |
| David | Hwang | david.hwang@yale.edu | United States of America | |
| Yousif | Jafar | Yousifjafar4@gmail.com | United States of America | |
| Michael | James | michael.james@duke.edu | United States of America | |
| Liz | Juma | lizjuma9656@gmail.com | Kenya |  |
| Cherry | Junn | cjp42@uw.edu | United States of America | |
| D. Ethan | Kahn | d.ethan.kahn@nyulangone.org | United States of America | |
| THEODORA | KALATZI | kalatzi.dora@gmail.com | United States of America | |
| Mariel | Kalkach Aparicio | kalkach@neurology.wisc.edu | Mexico |  |
| Lalit | Kaltenbach | lalit.kaltenbach@i-med.ac.at | Austria |  |
| Sujatha | Kannan | skannan3@jhmi.edu | United States of America | |
| Ata Murat | Kaynar | kaynarm@upmc.edu | United States of America | |
| Jessica | Kelemen | jkelemen@mgh.harvard.edu | United States of America | |
| Isaac | Kelleher-Unger | ik362@cam.ac.uk | United Kingdom |  |
| Fran | Kerrigan | fran.kerrigan@gmail.com | United States of America | |
| Imad | Khan | imad_khan@urmc.rochester.edu | United States of America | |
| Selam | Kifelew Melkamu | Selamjes@gmail.com | Ethiopia |  |
| Jennifer | Kim | jennifer.a.kim@yale.edu | United States of America | |
| Keri | Kim | skim42@uic.edu | United States of America | |
| Nerissa U. | Ko | nerissa.ko@ucsf.edu | United States of America | |
| Thomas | Kodankandath | kodankandath@gmail.com | United States of America | |
| Tomasz | Komendzinski | tkomen@umk.pl | Poland |  |
| Daniel | Kondziella | daniel_kondziella@yahoo.com | Denmark |  |
| Christopher | Kramer | ckramer1@neurology.bsd.uchicago.edu | United States of America | |
| Daniel | Krasna | krasnada@einstein.edu | United States of America | |
| Erik | Kulstad | erik.kulstad@utsouthwestern.edu | United States of America | |
| Abhay | Kumar | abhay.kumarjha@gmail.com | United States of America | |
| Myriam | LaCerte | myriamlacerte@yahoo.ca | United States of America | |
| Morgan | LaHolt | mlaholt@madonna.org | United States of America | |
| DENCHAI | LAIWATTANA | dlaiwattana@hotmail.com | Thailand |  |
| Adam | Lamm | adam.lamm@maryfreebed.com | United States of America | |
| Kevin | Law | kevin.law@florey.edu.au | Australia |  |
| Christos | Lazaridis | lazaridis@uchicago.edu | United States of America | |
| Rebecca | Lazeration | rebecca.lazeration@roseliassociates.com | United States of America | |
| Andy | Lee | andyl17@uci.edu | United States of America | |
| Ya-Han | Lee | 107003@w.tmu.edu.tw | Taiwan |  |
| ABHIJIT | Lele | abhijit2@uw.edu | United States of America | |
| Geoffrey | Ling | GLING1@JHMI.EDU | United States of America | |
| Eric | Liotta | Eric.liotta@northwestern.edu | United States of America | |
| Alexander | Liu | alliu1243@gmail.com | United States of America | |
| Sarah | Livesay | Sarah_l_livesay@rush.edu | United States of America | |
| Andrea | Loggini | andrea.loggini@uchospitals.edu | United States of America | |
| george | lopez | glopezmdphd@gmail.com | United States of America | |
| Marlina | Lovett | melovett@gmail.com | United States of America | |
| Dian | Lu | dl577@cam.ac.uk | United Kingdom |  |
| Andrea | Luppi | al857@cam.ac.uk | United Kingdom |  |
| Evan | Lutkenhoff | lutkenhoff@ucla.edu | United States of America | |
| Christine | MacDonald | cmacd@uw.edu | United States of America | |
| Carolina | Maciel | carolina.maciel@neurology.ufl.edu | United States of America | |
| Lori | Madden | lkmadden@ucdavis.edu | United States of America | |
| Chiara | Maffei | cmaffei@mgh.harvard.edu | United States of America | |
| Sherif | Mahmoud | smahmoud@ualberta.ca | Canada |  |
| Shraddha | Mainali | shraddha.mainali@osumc.edu | United States of America | |
| Trudy | Mallinson | trudy@gwu.edu | United States of America | |
| Miguel | Mancao | mmjmancao@gmail.com | United States of America | |
| Allie | Mandel | amandel@neurocriticalcare.org | United States of America | |
| Shirin | Mansooridara | sdara777@gmail.com | United States of America | |
| Ali | Mansour | Ali.mansour@uchospitals.edu | United States of America | |
| Rong | Mao | rong.mao@wisc.edu | United States of America | |
| Michael | Marino | marino01@einstein.edu | United States of America | |
| Charlotte | Martial | cmartial@uliege.be | Belgium |  |
| Candice | Martin | candicemartincaptioner@gmail.com | United States of America | |
| ROSE | MARUJO | rmarujo@mgh.harvard.edu | United States of America | |
| Charlotte | Maschke | Charlotte.Maschke@mail.mcgill.ca | Canada |  |
| Maryam | Masood | mmasood@mgh.harvard.edu | United States of America | |
| Janet | Masters | masterj@sutterhealth.org | United States of America | |
| Beril | Mat | beril8mat@gmail.com | Turkey |  |
| Subin | Mathew | Smathew@mgh.harvard.edu | United States of America | |
| Kelly | Matsudaira | Yeekm@uci.edu | United States of America | |
| Stephan | Mayer | stephanamayer@gmail.com | United States of America | |
| Aaron | McCabe | amccabe@minnetronixmedical.com | United States of America | |
| Victoria | McCredie | victoria.mccredie@uhn.ca | Canada |  |
| Mary | McMahon | mary.mcmahon@cchmc.org | United States of America | |
| Molly | McNett | mcnett.21@osu.edu | United States of America | |
| Fidel | Meira | fidelmeira@neurologista.org | Brazil |  |
| Jorge | Mejia-Mantilla | jorge.mejia.m@me.com | Colombia |  |
| Kara | Melmed | kara.melmed@gmail.com | United States of America | |
| Carolina | Mendoza-Puccini | carolina.mendoza-puccini@nih.gov | United States of America | |
| David | Menon | dkm13@cam.ac.uk | United Kingdom |  |
| William | Meurer | wmeurer@med.umich.edu | United States of America | |
| Maramelia | Miranda Alves | maramelia.miranda@unifesp.br | Brazil |  |
| Dick | Moberg | dick@moberg.com | United States of America | |
| elena | monai | elena24mn@gmail.com | United States of America | |
| Martin M | Monti | mmonti@mednet.ucla.edu | United States of America | |
| Jayaji | More | jmore@student.nymc.edu | United States of America | |
| Megan | Moyer | megantmoyer@gmail.com | United States of America | |
| Susanne | Muehlschlegel | susanne.muehlschlegel@umassmemorial.org | United States of America | |
| Malissa | Mulkey | mamulk@iu.edu | United States of America | |
| Marina | Munar | marina.munari@aopd.veneto.it | Italy |  |
| Hatem | Murad | hatem_murad@yahoo.com | Israel |  |
| Brooke | Murtaugh | bmurtaugh@madonna.org | United States of America | |
| Danielle | Nadin | danielle.nadin@mail.mcgill.ca | Canada |  |
| Masao | Nagayama | nagay001@iuhw.ac.jp | Japan |  |
| Christian | Nduka | ndkchris@gmail.com | Nigeria |  |
| Esther | Nemetsky | Enemetsky.bina@gmail.com | United States of America | |
| Linh | Nguyen | linh.nguyen@stlcop.edu | United States of America | |
| Masachika | Niimi | pomardon2010@gmail.com | Belgium |  |
| Christa O’Hana | Nobleza | christaohana14md@yahoo.com | United States of America | |
| Filipa | Noronha Falcão | fnoronhafalcao@gmail.com | Portugal |  |
| Pamela | Nye | pamelajanenye@neurosciencenursing.org | United States of America | |
| Paul | Nyquist | Pnyquis1@jhmi.edu | United States of America | |
| Susan | O'Connor | susan.jane.oconnor@gmail.com | United States of America | |
| John | O'Donnell | john.charles.odonnell@gmail.com | United States of America | |
| DaiWai | Olson | DaiWai.Olson@UTSouthwestern.edu | United States of America | |
| Kristine | O'Phelan | kophelan@med.miami.edu | United States of America | |
| Claude | Oster | coster@compuserve.com | United States of America | |
| Adrian | Owen | uwocerc@uwo.ca | Canada |  |
| Sandra | Pacheco Noriega | sakapan@yahoo.com | Spain |  |
| Juan | Padilla | juanmpadilla@hotmail.com | United States of America | |
| Rajanikant | Panda | rajanikant.panda@uliege.be | Belgium |  |
| Gunjan | Parikh | gparikh@som.umaryland.edu | United States of America | |
| Soojin | Park | gparikh@som.umaryland.edu | United States of America | |
| Tatiana | Pasternak | tatiana.pasternak@nih.gov | United States of America | |
| Viren | Patel | vdpatel5@gmail.com | United States of America | |
| Alexander | Peattie | sp3291@cumc.columbia.edu | United Kingdom |  |
| Mary | Pelleymounter | ardp2@cam.ac.uk | United States of America | |
| Andrew | Peterson | mary.pelleymounter@nih.gov | United States of America | |
| Kathleen | Pierson | apeter31@gmu.edu | United States of America | |
| Shanti | Pinto | kpierson@neuroptics.com | United States of America | |
| Pirouz | Piran | shanti.pinto@atriumhealth.org | United States of America | |
| Michael | Pizzi | michael.pizzi@neurology.ufl.edu | United States of America | |
| len | Polizzotto | len_polizzotto@yahoo.com | United States of America | |
| Sebastian | Pollandt | sebastian_pollandt@rush.edu | United States of America | |
| Phylisia | Pratt | phylisia.pratt@gmail.com | Saudi Arabia |  |
| Luis | Prera | luisprera@outlook.com | Guatemala |  |
| Lara | Prisco | lara.prisco@ouh.nhs.uk | United Kingdom |  |
| Relfa | Proano | mdrelfapp@hotmail.com | United States of America | |
| Jose Javier | Provencio | jp3b@virginia.edu | United States of America | |
| Louis | PUYBASSET | louis.puybasset@aphp.fr | France |  |
| Shireen | Qureshi | Shireen.alqureshi@gmail.com | Saudi Arabia |  |
| Shanta | Rajaram | shanta.rajaram@nih.gov | United States of America | |
| Courtney | Real | creal@mednet.ucla.edu | United States of America | |
| Ranier | Reyes | ranier.reyes@utsouthwestern.edu | United States of America | |
| Richard | Riker | rriker@cmamaine.com | United States of America | |
| Bridget | Rizik | brizik@partners.org | United States of America | |
| Daniel | Rodrigues | danielfdebarros@hotmail.com | Brazil |  |
| Ashley | Rogers | ashleyrogersmd@gmail.com | United States of America | |
| Toya | Rogers | toya.rogers@nih.gov | United States of America | |
| Benjamin | Rohaut | benjamin.rohaut@aphp.fr | France |  |
| Alison | Roomsburg | Alison.Roomsburg@dignityhealth.org | United States of America | |
| Fernando | Roosemberg Ordóñez | fernando_roosemberg1@hotmail.com | Ecuador |  |
| Eric | Rosenthal | erosenthal@mgh.harvard.edu | United States of America | |
| Daniel | Rubin | drubin4@partners.org | United States of America | |
| JoMarie | Rusche | jomarie.rusche@va.gov | United States of America | |
| Sergey | Ryzhov | sryzhov@mmc.org | United States of America | |
| Arianna | Sala | ariannasala@gmail.com | Italy |  |
| Daniel | Samano | dxs1059@med.miami.edu | United States of America | |
| Gisele | Sampaio | giselesampaio@hotmail.com | Brazil |  |
| William | Sanders | wsanders@mgh.harvard.edu | United States of America | |
| Melissa | Sandler | sandlerm@vcu.edu | United States of America | |
| Leandro | Sanz | leandro.sanz@uliege.be | Belgium |  |
| Gemmalynn | Sarapuddin | gbsarapuddin@gmail.com | Philippines |  |
| Simone | Sarasso | simone.sarasso@unimi.it | Italy |  |
| Saman | Sargolzaei | Ssargolz@utm.edu | United States of America | |
| Mica | Schachter | Micaela.fts@gmail.com | United States of America | |
| Nicholas | Schiff | nds2001@med.cornell.edu | United States of America | |
| Caroline | Schnakers | cschnakers@casacolina.org | United States of America | |
| David | Seder | sederd@mmc.org | United States of America | |
| Dmitry | Sergeev | dmsergeev@yandex.ru | Russian Federation | |
| Vishank | Shah | vshah2@uams.edu | United States of America | |
| Kartavya | Sharma | kartavya.sharma@utsouthwestern.edu | United States of America | |
| Tarek | Sharshar | tsharshar@gmail.com | France |  |
| Samer | Shoshan | samershoshan@icloud.com | United Arab Emirates | |
| Maryum | Shoukat | shoukatmaryum70@gmail.com | United States of America | |
| Lori | Shutter | shutterla@upmc.edu | United States of America | |
| Alireza | Sibaei | alisibaei@yahoo.de | Germany |  |
| Gordon | Siu | gordon.siu@midmichigan.org | United States of America | |
| Rebecca | Smith | Bjsmith@ascension.org | United States of America | |
| wade | smith | wade.smith@ucsf.edu | United States of America | |
| Sam | Snider | ssnider@partners.org | United States of America | |
| Lennart R B | Spindler | lrbs2@cam.ac.uk | United Kingdom |  |
| Marilyn | Spivack | mspivack@partners.org | United States of America | |
| Emmanuel | Stamatakis | eas46@cam.ac.uk | United Kingdom |  |
| Robert | Stevens | rstevens@jhmi.edu | United States of America | |
| Jesse | Stover | jstover@moberg.com | United States of America | |
| Jose | Suarez | jsuarez5@jhmi.edu | United States of America | |
| Gene | Sung | gsung@usc.edu | United States of America | |
| Lauren | Sutton | lauren.sutton@bjc.org | United States of America | |
| Christine | Swanson-Fischer | christine.swanson-fischer@nih.gov | United States of America | |
| Dionne | Swor | dswor@wakehealth.edu | United States of America | |
| Dorottya | Szocs | dorottya.szocs18@gmail.com | Romania |  |
| Gonzalo | Tamayo | gonzalotamayo@gmail.com | Spain |  |
| Carol | Taylor-Burds | carol.taylor-burds@nih.gov | United States of America | |
| MARCOS AURELIO | TEHERAN WILCHES | sepsis_1001@hotmail.com | Argentina |  |
| Robert Jayson | Tejam | jaysontejam@gmail.com | Philippines |  |
| Aurore | Thibaut | athibaut@uliege.be | Belgium |  |
| Zachary | Threlkeld | zthrelk@stanford.edu | United States of America | |
| Michel | Torbey | mtorbey@salud.unm.edu | United States of America | |
| Miriam | Treggiari | miriam.treggiari@yale.edu | United States of America | |
| Reese | Triana | rxt80@miami.edu | United States of America | |
| Preet | Varade | preet_m.varade@lvhn.org | United States of America | |
| Marko | Vasic | marko.vasic@jhsmiami.org | United States of America | |
| Vigneswaran | Veeramuthu | vicveera@gmail.com | Malaysia |  |
| Chethan | Venkatasubba Rao | cprao@bcm.edu | United States of America | |
| Paul | Vespa | pvespa@mednet.ucla.edu | United States of America | |
| Walter | Videtta | wvidetta@gmail.com | Argentina |  |
| Pitchamol | Vilaisaktipakorn | Pitchamol.vilaisaktipakorn@uchospitals.edu | United States of America | |
| Willa | Vo | willa.vo@utsouthwestern.edu | United States of America | |
| Amy | Wagner | wagnerak@upmc.edu | United States of America | |
| Mark | Wainwright | mwa110@uw.edu | United States of America | |
| Malaika | Walton | mwalton@infinityconferences.com | United States of America | |
| Jing | Wang | jingwangwyl@gmail.com | United States of America | |
| Tyler | Warren | tyler.warren@umassmemorial.org | United States of America | |
| Jennifer | Weaver | jenweaver524@gwu.edu | United States of America | |
| John | Whyte | jwhyte@einstein.edu | United States of America | |
| Bonny | Wong | bonnysuwong@gmail.com | United States of America | |
| Jean | Woo | jeaneugenepark@gmail.com | Korea, Republic of |  |
| Bill | Worthen | bill@neuroptics.com | United States of America | |
| Aleksandra | Yakhkind | sashkind@gmail.com | United States of America | |
| Teddy | Youn | teddy.youn@barrowneuro.org | United States of America | |
| Michael | Young | michael.young@mgh.harvard.edu | United States of America | |
| Ross | Zafonte | RZafonte@mgh.harvard.edu | United States of America | |
| Setareh | Zandi | szandiha@uci.edu | United States of America | |
| Yared | Zewde | Yaredzene121@gmail.com | Ethiopia |  |
| Bei | Zhang | beizhangmd@gmail.com | United States of America | |
| Ting | Zhou | Ting.zhou@nyulangone.org | United States of America | |
| Wendy | Ziai | weziai@jhmi.edu | United States of America | |
| Elizabeth | Zink | ezink1@jhmi.edu | United States of America | |
| Laura | Zitella Verbick | lverbick@minnetronixmedical.com | United States of America | |
| Prem |  | nattanmaip@health.missouri.edu | United States of America | |
