# Supplementary material for: Proceedings of the First Curing Coma Campaign NIH Symposium: Challenging the Future of Research for Coma and Disorders of Consciousness
Source: Neurocrit Care. 2021 Jul 8;35(Suppl 1):4–23. doi: 10.1007/s12028-021-01260-x (PMC8264966; doi:10.1007/s12028-021-01260-x)
Supplement: Supplementary file 2 — Supplementary file2 (PDF 581 kb) [file 12028_2021_1260_MOESM2_ESM.pdf]

Curing Coma: challenging the future of research for coma and disorders of consciousness
September 9-10, 2020
Part I. NIH Virtual Meeting

Purpose:
To bring together a diverse and multidisciplinary group of individuals (scientists, clinician-scientists, patient advocacy representatives, and industry partners) to optimize and accelerate the science of recovery of consciousness. Curing Coma is a challenge that is broadly defined by the goal to **promote recovery of consciousness through early intervention and long-term support**.

Attendee Engagement:
Throughout these 2 days, attendees are asked to write down research priorities for each of six topics. These priorities will be collated and assigned to the working groups (WG) mentioned below. The WG will continue developing the assigned priorities within the context of what has already been accomplished, and present recommendations during the *in-person meeting (Part II) May 3, 2021 at the NIH Natcher Conference Center in Bethesda, MD, USA*. Virtual Meeting attendees will be encouraged to sign up for one of the six WGs.

1. Research priorities for Biology of Coma
    i. facilitator: J Javier Provencio
2. Research priorities for Coma Database
    i. Facilitator: Paul Vespa
3. Research priorities for Care of the Comatose patient
    i. Facilitator: Molly McNett
4. Research priorities for Prognostication
    i. Facilitator: Jan Claassen
5. Research priorities for Early Clinical Trials
    i. Facilitator: Lori Shutter
6. Research priorities for long-term recovery
    i. Facilitator: Amy Wagner

Planning Committee
- NINDS: 3-4 + other NIH investigators
- NCS:
    - Melanie Boly, MD, PhD (University of Wisconsin)
    - J Claude Hemphill, MD (University of California, San Francisco)
    - Molly M McNett, RN, PhD (Ohio State University)
    - DaiWai M Olson, RN, PhD (UT Southwestern)
    - Jose Javier Provencio, MD (University of Virginia)
    - Lori Shutter, MD (University of Pittsburgh)
    - Jose I Suarez, MD (Johns Hopkins University)
    - Paul Vespa, MD (University of California, Los Angeles)

Session format

5 min	Why is this relevant to curing coma?
20 min	Content / State of the science
5 min	What is the Gap?

Panel discussion format

25 min	Two facilitators will help moderate these open discussions, which will consist of questions from attendees for the panel of investigators that prepared the content of the lecture.

Day 1. **Wednesday. 9 Sept 2020.**

Time	Item Speaker	Moderator
10:00 – 10:30	Opening – Meeting Objectives	Jeremy Brown<br>Jose I Suarez<br>J Claude Hemphill
10:45 – 11:15	Session 1 Defining phenotypes<br>Robert Stevens *<br>Michael Diringer<br>Geert Meyfroidt<br>Eric Rosenthal	Co-Chairs:<br>Javier Provencio<br>Shraddha Mainali
11:15 – 11:40	Panel Discussion	Q&A Facilitators:<br>Lori Madden<br>Holly Hinson
11:40 – 11:45	Summarize	Javier Provencio<br>Shraddha Mainali
11:45 – 12:00	Break	
12:00 - 12:30	Session 2: Biomarkers<br>Brian Edlow *<br>Simone Sarasso<br>Sherry Chou<br>Nicholas Schiff	Co-Chairs:<br>Sheila Alexander<br>Melanie Boly
12:30 – 12:55	Panel Discussion	Q&A Facilitator<br>Paul Nyquist<br>Karen Hirsch
12:55 - 1:00	Summarize	Sheila Alexander<br>Melanie Boly
1:00 – 1:15	Break	
1:15 – 1:45	Session 3: Proof-of-concept clinical trials<br>Lori Shutter *<br>Daniel Kondziella<br>Stephan Mayer<br>Dan Hanley<br>Olivia Gosseries	Co-Chairs:<br>Molly McNett<br>Yama Akbari
1:45 – 2:10	Panel Discussion	Q&A Facilitators:<br>Wendy Ziai<br>Santosh Murthy
2:10 - 2:15	Summarize	Molly McNett<br>Yama Akbari
2:15 – 2:30	Closing	

*Each session will brainstorm ahead of time and pick a speaker or two to present

Day 2. **Thursday. 10 Sept 2020.**

Time	Item	Speaker
10:00 – 10:30	Session 4. Neuroprognostication<br>Wendy Ziai *<br>Brandon Foreman<br>Tom Bleck<br>David Greer<br>Susanne Muehlschlegel	Co-Chairs: 2<br>Jed Hartings<br>Theresa Human
10:30 – 10:55	Panel Discussion	Q&A Facilitators:<br>Sarah Livesay<br>Elizabeth Zink
10:55 – 11:00	Summarize	Jed Hartings<br>Theresa Human
11:00 – 11:15	Break	
11:15 – 11:45	Session 5. Long-term Recovery<br>Kathleen Bell *<br>Theresa Green<br>John Whyte<br>Martin Monti	Co-Chairs<br>Joseph Giacino<br>Amy Wagner
11:45 - 12:10	Panel Discussion	Q&A Facilitators:<br>MaryKay Bader<br>Nerissa Ko
12:10 – 12:15	Summarize	Joseph Giacino<br>Amy Wagner
12:15 – 12:30	Break	
12:30 – 1:00	Session 6. Large Data Sets (Database)<br>Luke James *<br>Raimund Helbok<br>Tarek Sharshar<br>Jose Suarez	Co-Chairs:<br>Paul Vespa<br>Victoria McCredie
1:00 – 1:25	Panel Discussion	Q&A Facilitators:<br>Walter Videtta<br>Gisele Sampaio
1:25 – 1:30	Summarize	Paul Vespa<br>Victoria McCredie
1:30 – 1:45	CLOSING Remarks	Jeremy Brown<br>Jose I Suarez<br>J Claude Hemphill

*Each session will brainstorm ahead of time and pick a speaker or two to present
